# The effects of traditional, superset, and tri-set resistance training structures on perceived intensity and physiological responses

**DOI:** 10.1007/s00421-017-3680-3

**Published:** 2017-07-11

**Authors:** Jonathon J. S. Weakley, Kevin Till, Dale B. Read, Gregory A. B. Roe, Joshua Darrall-Jones, Padraic J. Phibbs, Ben Jones

**Affiliations:** 1 Room G03, Macaulay Hall, Institute for Sport, Physical Activity and Leisure, Centre for Sports Performance, Headingley Campus, Leeds Beckett University, West Yorkshire, LS6 3GZ UK; 2 Yorkshire Carnegie Rugby Union Club, Kirkstall Training Ground, Leeds Rugby Academy, Leeds, West Yorkshire UK; 3 The Rugby Football League, Leeds, West Yorkshire UK

**Keywords:** Efficiency, Resistance training, Countermovement jump, Testosterone, Cortisol, Lactate

## Abstract

**Purpose:**

Investigate the acute and short-term (i.e., 24 h) effects of traditional (TRAD), superset (SS), and tri-set (TRI) resistance training protocols on perceptions of intensity and physiological responses.

**Methods:**

Fourteen male participants completed a familiarisation session and three resistance training protocols (i.e., TRAD, SS, and TRI) in a randomised-crossover design. Rating of perceived exertion, lactate concentration ([Lac]), creatine kinase concentration ([CK]), countermovement jump (CMJ), testosterone, and cortisol concentrations was measured pre, immediately, and 24-h post the resistance training sessions with magnitude-based inferences assessing changes/differences within/between protocols.

**Results:**

TRI reported *possible* to *almost certainly* greater efficiency and rate of perceived exertion, although session perceived load was *very likely* lower. SS and TRI had *very likely* to *almost certainly* greater lactate responses during the protocols, with changes in [CK] being *very likely* and *likely* increased at 24 h, respectively. At 24-h post-training, CMJ variables in the TRAD protocol had returned to baseline; however, SS and TRI were still *possibly* to *likely* reduced. *Possible* increases in testosterone immediately post SS and TRI protocols were reported, with SS showing *possible* increases at 24-h post-training. TRAD and SS showed *almost certain* and *likely* decreases in cortisol immediately post, respectively, with TRAD reporting *likely* decreases at 24-h post-training.

**Conclusions:**

SS and TRI can enhance training efficiency and reduce training time. However, acute and short-term physiological responses differ between protocols. Athletes can utilise SS and TRI resistance training, but may require additional recovery post-training to minimise effects of fatigue.

## Introduction

Resistance training is known to improve measures of strength, power, and lean body mass (Pareja-Blanco et al. 2016). Furthermore, it is established that it can enhance physical performance (e.g., jump height) which may benefit sporting outcomes (Pareja-Blanco et al. 2016). However, athletes are often unable to commit prolonged periods of time to resistance training due to other training requirements (e.g., skill development and other conditioning priorities) (Phibbs et al. 2017). Therefore, resistance training protocols such as supersets (SS) (i.e., the completion of two exercises consecutively followed by a recovery period) and tri-sets (TRI) (i.e., the completion of three exercises consecutively followed by a recovery period) that enhance training efficiency (i.e., kilograms lifted per minute) may be an effective mechanism to provide an appropriate resistance training stimulus, in a short period of time (Sabido et al. 2016; Schoenfeld 2011).

The use of SS and TRI has been proposed as an efficacious method of enhancing strength and body composition (Robbins et al. 2010a, b). In studies that have investigated these training protocols, there does not appear to be any detrimental effect on resistance training volume despite the reduction in recovery time within each training session (Maia et al. 2014; Robbins et al. 2010a). However, this reduction in training time may alter perceptions of internal training load [i.e., rating of perceived exertion (RPE)] (Balsamo et al. 2012). To this end, SS and TRI can improve training efficiency, reduce training time, and alter perceived training load, although the magnitude of these changes is still unknown.

In addition to reduced training time and altered perception of intensity, resistance training methods that have varying rates of efficiency are also known to promote divergent metabolic and endocrine responses (Hiscock et al. 2017; Schoenfeld 2010; Walker et al. 2011). It has been suggested that the cause of this variation is in part a result of the resistance training efficiency imposed (McCaulley et al. 2009). It has been postulated that protocols that complete large amounts of volume within a given time elicit greater metabolic responses (Hooper et al. 2017). This increase in metabolic perturbation, evidenced by lowered pH and higher lactate concentrations ([LAC]), may then provoke augmented endocrine outcomes (i.e., increased testosterone and cortisol secretion) (Hooper et al. 2017). While previous research has examined relationships between resistance training efficiency, and metabolic and endocrine responses (Hiscock et al. 2017; McCaulley et al. 2009), the effect of enhanced efficiency due to SS and TRI resistance training structuring has not been considered. To this end, the physiological responses due to these training methods are not well understood. Furthermore, changes in training efficiency are also known to affect neuromuscular function (Hiscock et al. 2017). McCaulley et al. (2009) previously suggested that increased resistance training efficiency may cause a reliance upon anaerobic glycolysis and an accumulation of metabolites (i.e., [LAC]) which may damage contractile properties within the muscle (McCaulley et al. 2009). These changes may potentially cause reduced force-generating capacity and impact upon subsequent neuromuscular performance [e.g., countermovement jump (CMJ)]. However, volume and intensity controlled trials have not assessed the magnitude and short-term (i.e., 24 h) effects of increased training efficiency upon neuromuscular responses.

Establishing resistance training protocols and structures that effectively utilise an athlete’s time is of benefit. SS and TRI have previously been established as two forms of resistance training that manage this (Robbins et al. 2010a). However, the acute and short-term physiological responses to these methods of resistance training have not yet been established. Consequently, investigation of these training methods will provide an improved understanding of how enhanced training efficiency can affect perception of training load, metabolic and muscle damage outcomes, endocrine responses, and neuromuscular function following different resistance set structures. Therefore, the aim of this study was to investigate the acute and short-term (i.e., 24 h) effects of traditional (TRAD), SS, and TRI volume and intensity equated resistance training protocols on perception of training intensity, and metabolic, neuromuscular, and endocrine responses in well-trained male athletes.

## Methods

### Participants

Fourteen male university rugby union players with a resistance training history of over 2 years were recruited to take part in the study (Table 1). All participants had at least 6 months uninterrupted resistance training, performing at least three resistance training sessions each week. Participants were screened prior to the study for any contraindication to physical activity. Throughout the study participants refrained from dietary supplements, and the only medication used by one participant was for the treatment of mild asthma. All protocols were explained and informed consent was obtained from all individual participants included in the study prior to the beginning of the study. Ethics approval was granted by the university ethics committee.

**Table 1:** Mean ± SD of participant anthropometric and physical characteristics

Age (years)	20.8 ± 1.2	Bench press 3RM (kg)	105.2 ± 15.2
Height (m)	1.81 ± 0.06	Romanian deadlift 3RM (kg)	143.2 ± 30.8
Body mass (kg)	87.3 ± 6.2	Dumbbell shoulder press 3RM (kg)	66.0 ± 8.6
Training age (years)	4.1 ± 1.2	Bent-over row 3RM (kg)	95.0 ± 14.5
Back squat 3RM (kg)	141.1 ± 31.9	Upright row 3RM (kg)	60.1 ± 6.9

### Experimental design

**Fig. 1:**
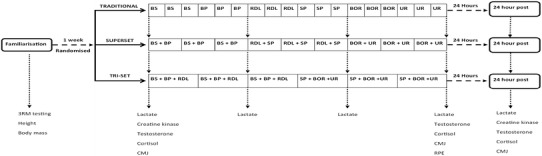
Diagram outlining the order of experimental procedures. *3RM* three repetition maximum, *CMJ* countermovement jump, *RPE* rate of perceived exertion, *BS* back squat, *BP* bench press, *RDL* Romanian deadlift, *SP* shoulder press, *BOR* bent-over row, *UR* upright row

This study was a randomised-crossover design that took place over 4 weeks at the beginning of the university rugby union pre-season (i.e., July–August). All participants had followed an off-season resistance training programme and initiated the study with a comparable training base. The study consisted of a familiarisation assessment, followed by three resistance training protocols (i.e., TRAD, SS, TRI) in a randomised order. Twenty-four hours after the resistance training protocol participants were required to return to the exercise laboratory for follow-up measures. Figure 1 further outlines the protocols and timepoints. In the 48 h before all testing, participants were asked to refrain from vigorous exercise, maintain normal dietary patterns, sleep well (i.e., >7 h), consume a meal 2 h before testing, and maintain normal hydration levels. After each testing session, participants were asked to consume their regular post-exercise meal and to not partake in any further exercise for 24 h.

### Resistance training protocols

Each protocol contained the same six exercises (i.e., back squat, bench press, Romanian deadlift, dumbbell shoulder press, bent-over row, and upright row), although grouped into a TRAD, SS, or TRI configuration (refer to Fig. 1). TRAD resistance training referred to the completion of a single exercise set followed by a rest period. All exercise sets were then completed prior to completing any other exercises. In the SS protocol, however, two different exercise sets were completed consecutively followed by a rest period. These two different exercises were then repeated until the required number of sets was completed. TRI involved an additional exercise and set (i.e., three consecutive sets of different exercises) followed by a rest period. All three exercise sets were then completed prior to completing the final three exercises. Sixty-five percent of each movement’s three repetition maximum (3RM) was calculated and used as the prescribed intensity for each exercise across the three training protocols. This intensity was selected due to previous research by Sabido et al. (2016) indicating that when completing supersets, intensities above this cause notable losses in repetition completion (i.e., 12.5%). All participants participated in the three resistance training sessions with exactly 7 days between protocols.

On testing days, participants were informed of the protocol that would be completed, and provided a salivary and finger-tip blood sample on arrival for the analysis of testosterone and cortisol concentration, and [Lac] and creatine kinase concentration [CK], respectively. Upon the completion of a standardised warm up, which included dynamic exercise and exercise specific stretches [i.e., walking lunges, squats, heel flicks, high knees, skipping, three submaximal CMJ, and plyometric push ups (Roe et al. 2016b)], a CMJ was completed upon a force platform (NMP Technologies Ltd., ForceDecks Model FD4000a, London, UK). After the CMJ, an exercise specific warm up utilising the first exercise/grouping of exercises (i.e., squat in the TRAD; squat and bench press in the SS; squat, bench press, and Romanian deadlift in the TRI) was completed. This consisted of eight repetitions with an empty bar, followed by two sets of five repetitions and then three repetitions at submaximal self-selected loads. This has previously been completed in resistance training literature (Darrall-Jones et al. 2015; Weakley et al. 2017a, b). After the completion of the exercise specific warm up, the exercises were organised with 65% of the participants 3RM and a 2-min rest prior to the commencement of exercise was provided.

Exercises were grouped, so that similar muscle groups were not completed consecutively (Sabido et al. 2016), and so that large muscle groups were exercised prior to smaller muscle groups (e.g., back squat followed by bench press) (Haff and Triplett 2015). The specific exercise routine was undertaken with 2-min rest between every exercise set/grouping of exercises. While all repetitions maintained a two second eccentric phase (monitored by the lead researcher) followed by an explosive concentric phase. In total, six exercises each consisting of three sets and ten repetitions were completed. Finger-tip blood samples were taken after the 6th and 12th sets for the assessment of [Lac]. At the completion of the 18th and final set, finger-tip samples were taken again (for [Lac]), along with CMJ and salivary sample (for testosterone and cortisol concentration). 15 min after the completion of each resistance training protocol participants provided an RPE utilising a modified-Borg scale (Singh et al. 2007). At 24-h post-protocol, participants returned and provided a 5 ml salivary sample and a finger-tip blood sample was taken for [CK]. Following these measures, participants completed the same standardised warm up as the previous day and completed CMJ testing upon the same force plate. Figure 1 outlines the protocols and timepoints of testing.

### Protocols

#### Strength measures and anthropometric assessment

During a familiarisation session 3RM strength, body mass, and height were measured. Body mass and height were measured to the nearest 0.1 kg and 0.1 cm, respectively, using calibrated Seca Alpha (model 220, Germany) scales and Seca Alpha (model 213, Germany) stadiometer. Strength was measured with the back squat, bench press, Romanian deadlift, dumbbell shoulder press, bent-over row, and upright row being tested. These movements were chosen as they have previously been used in similar studies (Harries et al. 2016; Smart and Gill 2013; Till et al. 2015), while all participants were familiar with the movements as they had been in previous resistance training programmes.

3RM strength was assessed using the following exercise protocols, previously outlined (Haff and Triplett 2015; Smart and Gill 2013; Uribe et al. 2010; Weakley et al. 2017b). The back squat was completed with the bar resting on the upper trapezium with participants required to lower themselves, so that the top of the thigh was parallel with the floor. The bench press was completed with hand position at a self-selected width with the bar lowered to the chest and returned to a locked-out position. Romanian deadlift maintained a slight bend in the knee and the lowering of the bar until immediately below the patellar. Dumbbell shoulder press begun with the arms holding the dumbbells, so that the elbow was at a 90° angle with the dumbbells in line with the cranium. The arms were extended and lowered, so that the arms returned to the 90° elbow angle. The bent-over row was completed with an overhand grip which raised the bar to the lower sternum, while the torso was maintained parallel to the ground. The upright row required the participant to hold the barbell with a shoulder width grip; the bar was then raised to the nipple line and returned to the hang position without any additional leg drive.

#### Hormone assessment

Saliva samples were taken at three timepoints (i.e., at arrival, immediately post the completion of each protocol, and at the 24-h post-protocol follow-up) across the 24-h period. To standardise collection of all salivary samples, participants were asked not to eat or brush their teeth within 60 min of providing a sample. Furthermore, 15 min before initiating a passive drool, participants were provided 200 ml of water. This was used to ensure minimal sample dilution and to remove food residue within the mouth. The first and last saliva samples were taken at the same time each day to assist in the control for circadian rhythm. Saliva was used due to its previous use in rugby union players, ease of compliance, low invasiveness, and ability to measure biologically active hormones (e.g., testosterone and cortisol) (Crewther and Cook 2010; Gaviglio et al. 2014).

A 5 ml volume of saliva was deposited from the mouth into a 10 ml cryovial and centrifuged within 60 min of provision. Centrifugation was at 3000 RPM for 15 min with the supernatant then being transferred by pipette to commercially available storage equipment (Salimetrics Cryovials, Salimetrics, CA, USA). This was then stored immediately at −80 °C prior to analysis at a private commercial laboratory (Psychology Laboratory, Anglia Ruskin University, UK). The interassay coefficient of variation (CV) for the hormone responses was reported to be 4.3% for testosterone and 7.2% for cortisol.

#### CMJ assessment

Analysis of CMJ was completed using a force platform (NMP Technologies Ltd., ForceDecks Model FD4000a, London, UK) which sampled at a rate of 1000 Hz. All participants performed three CMJs with feet placed approximately shoulder width apart and with hands placed on hips. Participants lowered themselves to a self-selected depth and jumped as high as possible. Between each maximal exertion, 60 s rest was provided (Weakley et al. 2017b). The outcome variables which were included in the analysis were: CMJ height (jump height), flight time:contraction time (FT:CT), and peak power per kg of body mass (PP/BM). Jump height was used due to its common use as a measure of lower body power (Roe et al. 2016b; Till et al. 2016; Weakley et al. 2017b). FT:CT was assessed as it provides the practitioner insight into movement strategies and has been suggested to be a valuable measure of fatigue due to it being derived from time-related variables (Gathercole et al. 2015; McGuigan 2017). PP/BM was analysed due to its very large relationship (*r* = 0.81) with ballistic capabilities (Hori et al. 2008). The best of the three scores at each timepoint was used in analysis (Haddad et al. 2015). Participant CV of these variables was reported to be 4.6% (jump height), 7.8% (FT:CT), and PP/BM (1.3%).

#### Metabolic assessment

Whole blood samples were collected to assess [CK] upon arrival at the testing facility and at the same corresponding timepoint 24 h later. Samples were collected via finger-tip puncture which was made with a spring-loaded single use disposable lancet. Approximately 30 µl of whole capillary blood was collected using a plastic capillary tube (MICROSAFE©, Safe-tec, Numbrecht, Ivyland, USA) and immediately analysed using reflectance photometry (Reflotron^®^ Plus, Boehringer, Manheim, Germany). 20 min before each testing session, the machine was calibrated using a standardised [CK] strip. Participant reliability of this machine has previously been reported (CV = 5.3%) (Roe et al. 2016a).

Blood [Lac] was analysed before, during, and after the exercise protocols using a lactate analyser (Lactate Plus, Nova Biomedical, MA, USA). After sterilising the finger, a puncture was made with a spring-loaded single use disposable lancet. The first drop of blood was wiped away, with the second drop being applied to an assay strip and inserted into the [LAC] analysing device. This device has previously been reported to demonstrate high levels of reliability (intraclass correlation coefficient = 0.99) at a range of [Lac] values (Baldari et al. 2009).

#### RPE and session perceived load measures

Participants were asked to provide an RPE 15 min after each resistance training protocol after being asked the question “How was your workout?” Participants were presented with a modified-Borg ratio-10 scale and verbally indicated an answer which was recorded (Singh et al. 2007). Training time was recorded to the nearest minute of duration by the lead researcher, with this time being recorded and then multiplied with the corresponding RPE value to provide session perceived load (Foster et al. 2001).

#### Calculation of volume load and efficiency

Volume load (kg) [i.e., the multiplication of all sets, repetitions, and weight (kg)] has previously been used as a means of calculating resistance training loads (Peterson et al. 2011). The volume load for each participant was standardised across each protocol, with all sets, repetitions, and external weight being used in the calculation. The volume load value was then divided by training duration in minutes (previously used in the calculation of session perceived load) to calculate training efficiency (kg min^−1^). This method of calculating resistance training efficiency has previously been used (Robbins et al. 2010a, c).

### Statistical analyses

Data are presented as mean ± standard deviation (SD) or standardised effect size (ES) ± 90% confidence intervals (90% CI). Prior to analysis, all data were log transformed to reduce bias arising from non-uniformity error, and then analysed for practical significance using magnitude-based inferences (Batterham and Hopkins 2006). The threshold for a change to be considered to meet the smallest worthwhile change/difference (SWC/D) was set at 0.2 × the between participant SD and was calculated using an online spreadsheet (Hopkins 2006b). For between group comparisons (e.g., TRAD vs. SS), the standardised ES of the variables being analysed were compared to assess the magnitude of difference between the two protocols (Hopkins 2006a). The probability that the magnitude of change or difference was greater than the SWC/D was rated as <0.5%, *almost certainly not*; 0.5–5%, *very unlikely*; 5–25%, *unlikely*; 25–75%, *possibly*; 75–95%, *likely*; 95–99.5%, *very likely*; >99.5%, *almost certainly* (Hopkins et al. 2009). Where the 90% confidence interval (CI) crossed both the upper and lower boundaries of the SWC/D (ES ± 0.2), the magnitude of change was described as *unclear* (Hopkins et al. 2009). ES thresholds were set at <0.2 (*trivial*), 0.2–0.6 (*small*), 0.6–1.2 (*large*), and 1.2–2.0 (*very large*) (Hopkins et al. 2009).

## Results

### Perceived exertion, volume load, and efficiency

The mean ± SD for time, RPE, session perceived load, and efficiency (kg min^−1^) of each protocol are presented in Table 2. In addition, inferences and ES (±90% CI) of between condition comparisons are supplied.

**Table 2:** Mean ± SD reported time, RPE, session perceived load, and efficiency, and between condition comparison of traditional (TRAD), superset (SS), and tri-set (TRI) resistance training protocols

	TRAD Mean ± SD	SS Mean ± SD	TRI Mean ± SD	TRAD vs. SS Inference ES ± 90% CI	SS vs. TRI Inference ES ± 90% CI	TRAD vs. TRI Inference ES ± 90% CI
Time (min)	42.3 ± 1.3	24.0 ± 1.2	17.7 ± 1.6	TRAD > SS *Almost certainly* 15.68 ± 0.67	SS > TRI *Almost certainly* 8.46 ± 0.84	TRAD > TRI *Almost certainly* 24.14 ± 1.11
RPE (AU)	4.4 ± 2.2	6.8 ± 1.2	7.6 ± 0.8	TRAD < SS *Almost certainly* 1.05 ± 0.36	SS < TRI *Possibly* 0.27 ± 0.21	TRAD < TRI *Almost certainly* 1.32 ± 0.43
Session perceived load (AU)	184.2 ± 92.1	163.1 ± 34.3	134.9 ± 17.7	TRAD > SS *Possibly* 0.28 ± 0.34	SS > TRI *Very likely* 0.72 ± 0.39	TRAD > TRI *Very likely* 0.44 ± 0.17
Efficiency (kg min^−1^)	275.0 ± 52.3	483.3 ± 106.3	656.5 ± 144.4	TRAD < SS *Almost certainly* 3.10 ± 0.13	SS < TRI *Almost certainly* 1.67 ± 0.16	TRAD < TRI *Almost certainly* 4.78 ± 0.22

*TRAD* traditional protocol, *SS* superset protocol, *TRI* tri-set protocol, *Time* average time in minutes to complete each protocol, *RPE* rate of perceived exertion of each protocol, *Efficiency* the mean number of kilograms lifted per minute, *Min* minutes, *Mean* *±* *SD* mean ± standard deviation, *ES* *±* *90%* *CI* standardised effect size ± 90% confidence interval

### Blood lactate concentration

**Fig. 2:**
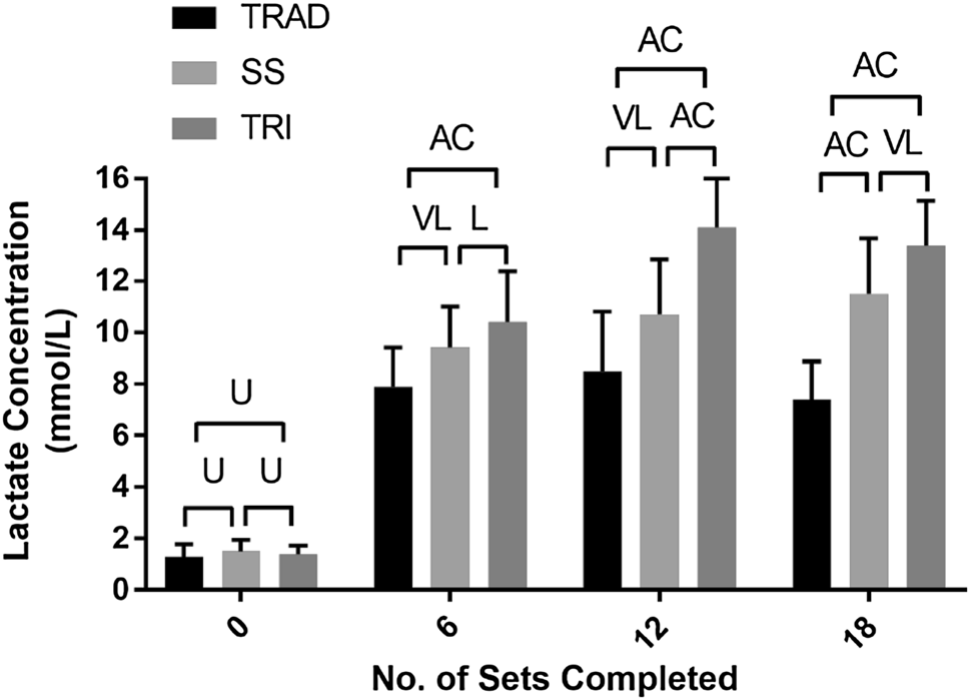
Blood lactate responses throughout a traditional, superset, or tri-set resistance training protocol. *U* unclear, *L* likely, *VL* very likely, *AC* almost certain

Figure 2 presents mean ± SD [Lac] and between group comparisons across the three protocols. [Lac] pre-exercise was: TRAD, 1.30 ± 0.50; SS, 1.50 ± 0.46; TRI, 1.40 ± 0.34 mmol l^−1^. At the completion of set 6 [Lac] was: TRAD, 7.90 ± 1.50; SS, 9.40 ± 1.60; TRI, 10.40 ± 1.98 mmol l^−1^. Between group comparisons showed *likely* to *almost certain* differences between protocols (ES ± 90% CI; TRAD vs. SS = 0.96 ± 0.51, SS vs. TRI = 0.59 ± 0.56, TRAD vs. TRI = 1.50 ± 0.51). At the completion of set, 12 [Lac] was: TRAD, 8.50 ± 2.30; SS, 10.70 ± 2.14; TRI, 14.10 ± 2.96 mmol l^−1^. Between group comparisons showed *very likely* to *almost certain* differences between protocols (ES ± 90% CI; TRAD vs. SS = 0.92 ± 0.60, SS vs. TRI = 1.42 ± 0.57, TRAD vs. TRI = 2.03 ± 0.51). Immediately, post-exercise [Lac] was: TRAD, 7.40 ± 1.48; SS, 11.50 ± 2.19; TRI, 13.40 ± 1.74 mmol l^−1^. Between group comparisons showed *very likely* to *almost certain* differences between protocols (ES ± 90% CI; TRAD vs. SS = 2.33 ± 0.74, SS vs. TRI = 0.84 ± 0.48, TRAD vs. TRI = 3.14 ± 0.54).

### Countermovement jump

**Fig. 3:**
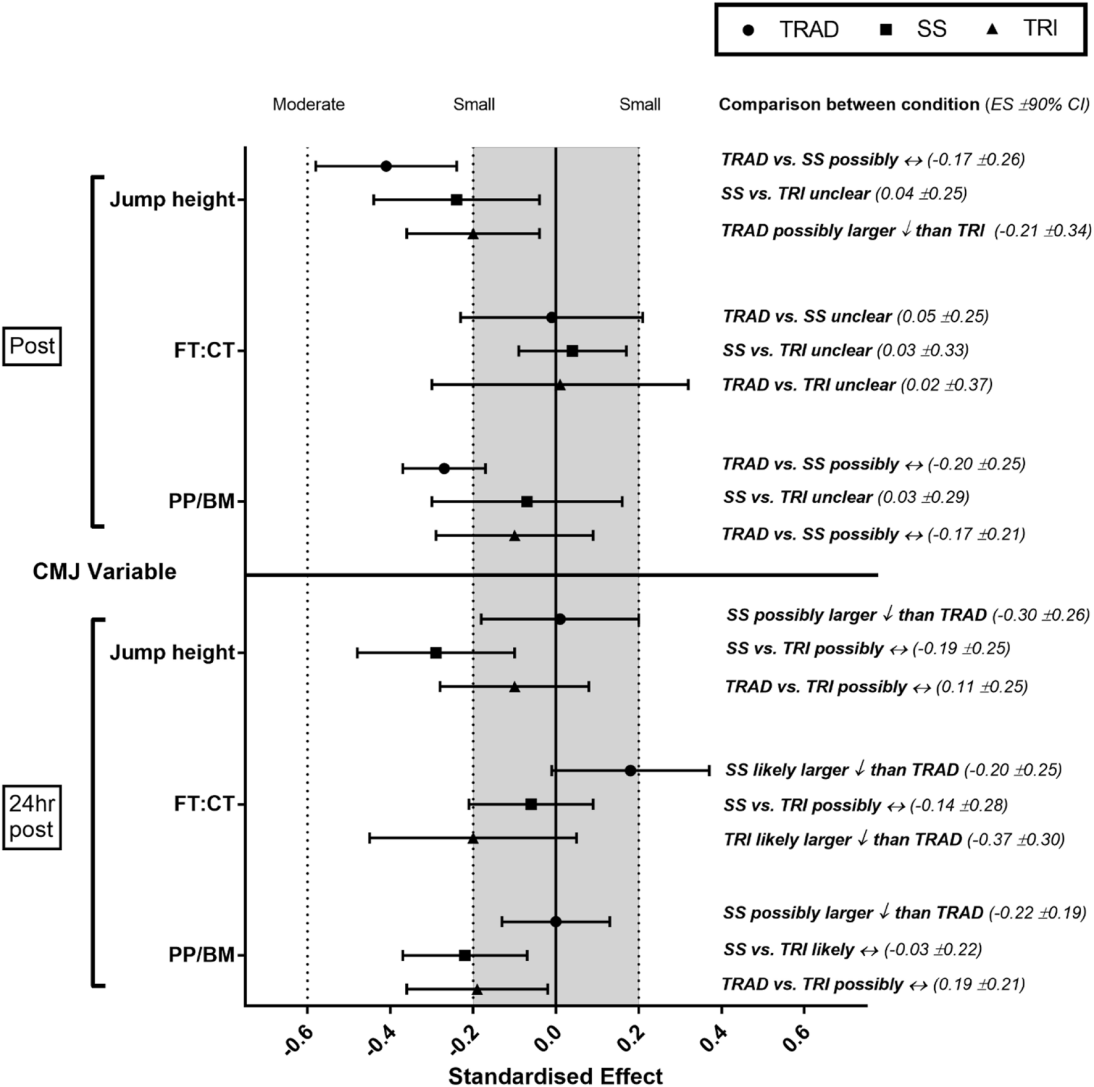
Standardised effect size (±90% CI) changes and inferences of between condition comparisons of CMJ variables immediately and 24 h after traditional (TRAD), superset (SS), and tri-set (TRI) resistance training

Table 3 presents the mean ± SD performance of each CMJ variable at pre-, post-, and 24-h post-exercise. Furthermore, percentage change (±90% CI), standardised ES (±90% CI), and inference of change between timepoints are provided. Figure 3 presents the standardised ES (±90% CI) change from pre- to post-exercise, and pre- to 24-h post-exercise. In addition, between condition comparisons of ES change are presented as ES (±90% CI) and inference.

**Table 3:** CMJ variable responses from pre-, post-, and 24-h post- a traditional (TRAD), superset (SS), and tri-set (TRI) resistance training protocol

	Pre- M ± SD	Post- M ± SD	Pre–Post %Δ ± 90% CI	Pre–Post ES ± 90% CI	Pre–Post Inference	Post 24 h- M ± SD	Pre–24 h %Δ ± 90% CI	Pre–24 h ES ± 90% CI	Pre–24 h Inference
TRAD									
Jump height (cm)	37.9 ± 6.1	35.5 ± 6.0	−6.2 ± 2.5	−0.41 ± 0.17	*Very Likely ↓*	37.9 ± 6.8	0.10 ± 3.0	0.01 ± 0.19	*Likely ↔*
FT:CT	0.65 ± 0.15	0.65 ± 0.17	−0.20 ± 4.9	−0.01 ± 0.22	*Unclear*	0.68 ± 0.14	4.1 ± 4.4	0.18 ± 0.19	*Possible ↑*
PP/BM (W kg^−1^)	55.3 ± 8.3	53.1 ± 7.4	−3.9 ± 1.5	−0.27 ± 0.10	*Likely ↓*	55.3 ± 8.3	0.0 ± 2.0	0.00 ± 0.13	*Very Likely ↔*
SS									
Jump height (cm)	38.9 ± 6.2	37.5 ± 5.6	−3.7 ± 3.1	−0.24 ± 0.20	*Possible ↓*	37.2 ± 6.0	−4.5 ± 2.9	−0.29 ± 0.19	*Likely ↓*
FT:CT	0.64 ± 0.15	0.65 ± 0.14	1.0 ± 3.1	0.04 ± 0.13	*Very Likely ↔*	0.63 ± 0.18	−1.3 ± 3.3	−0.06 ± 0.15	*Likely ↔*
PP/BM (W kg^−1^)	55.1 ± 6.6	54.7 ± 7.7	−0.90 ± 2.8	−0.07 ± 0.23	*Likely ↔*	53.7 ± 7.5	−2.7 ± 1.8	−0.22 ± 0.15	*Likely ↓*
TRI									
Jump height (cm)	38.3 ± 6.1	37.1 ± 6.7	−3.10 ± 2.4	−0.20 ± 0.16	*Possible ↓*	37.7 ± 6.4	−1.6 ± 2.7	−0.10 ± 0.18	*Likely ↔*
FT:CT	0.67 ± 0.14	0.67 ± 0.12	0.20 ± 6.3	0.01 ± 0.31	*Unclear*	0.65 ± 0.14	−3.9 ± 4.9	−0.20 ± 0.25	*Possible ↓*
PP/BM (W kg^−1^)	55.5 ± 10.0	54.5 ± 8.2	−1.8 ± 3.4	−0.10 ± 0.19	*Likely ↔*	53.6 ± 8.6	−3.3 ± 3.4	−0.19 ± 0.17	*Possible ↓*

*M* *±* *SD* mean ± standard deviation, *%Δ* percentage change, *ES* effect size, *90% CI* 90% confidence interval, *FT:CT* flight time:contraction time, *PP/BM* peak power per kilogram of body mass, *↑* increase, *↔* trivial, *↓* decrease

### Testosterone and cortisol concentration

**Fig. 4:**
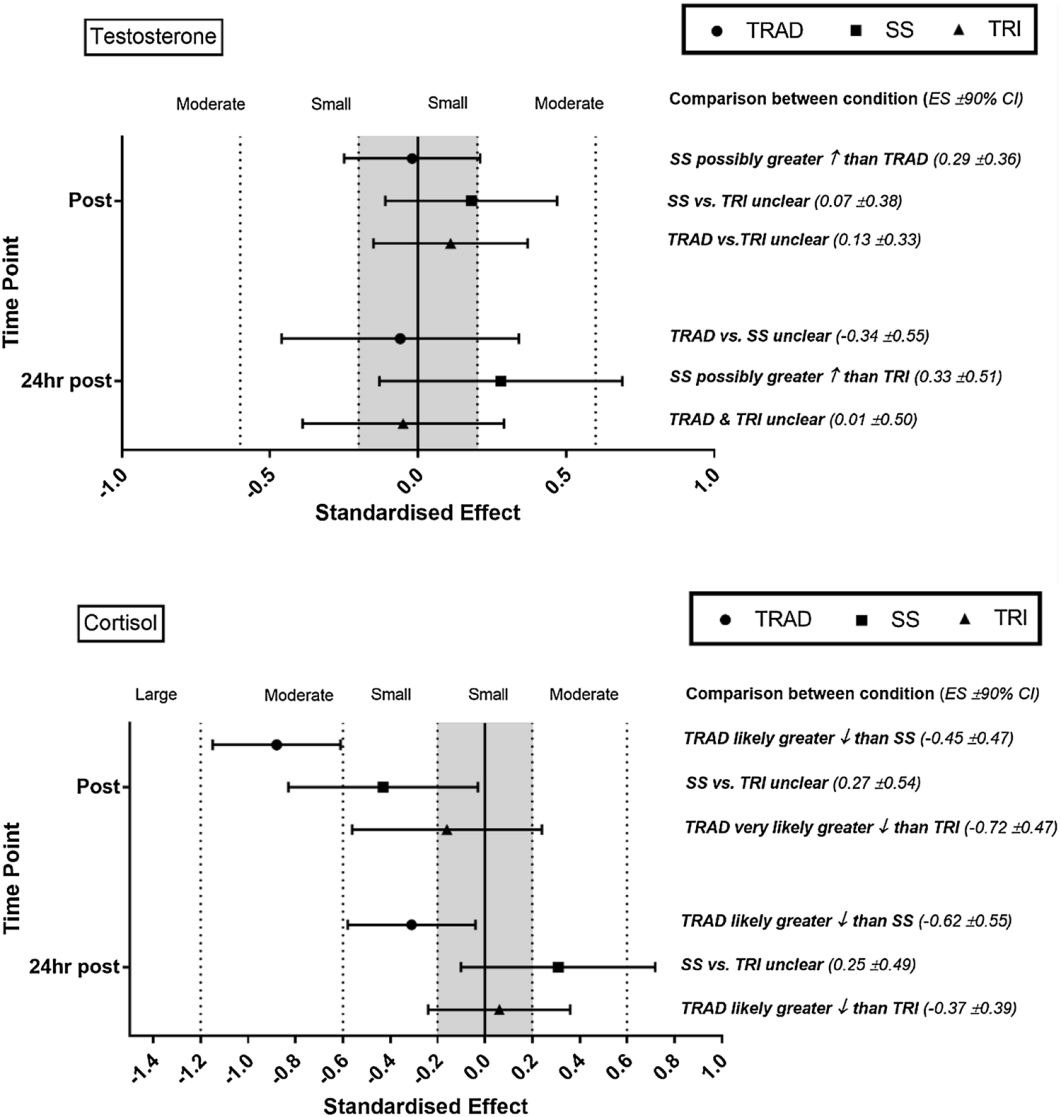
Standardised effect size (±90% CI) change of testosterone and cortisol immediately post- and 24 h after traditional (TRAD), superset (SS), and tri-set (TRI) resistance training. Also presented is the standardised effect size (±90% CI) and inference of comparisons between condition of testosterone and cortisol immediately and 24 h after exercise. ↓, decrease; ↑, increase

Table 4 presents mean ± SD mean value of testosterone and cortisol concentration at pre-, post-, and 24-h post-exercise. Furthermore, percentage change (±90% CI), standardised ES (±90% CI), and inference of change between timepoints are provided. Figure 4 presents the standardised ES (±90% CI) change from pre- to post-exercise, and pre- to 24-h post-exercise. In addition, between condition comparison of ES change is presented as ES (±90% CI) and inference.

**Table 4:** Testosterone (pg ml^−1^) and cortisol (ng ml^−1^) responses from pre-, post-, and 24-h post- a traditional (TRAD), superset (SS), and tri-set (TRI) resistance training protocol

	Pre- M ± SD	Post- M ± SD	Pre–Post %Δ ± 90% CI	Pre–Post ES ± 90% CI	Pre–Post Inference	Post 24 h- M ± SD	Pre–24 h %Δ ± 90% CI	Pre–24 h ES ± 90% CI	Pre–24 h Inference
Testosterone (pg ml^−1^)									
TRAD	139.6 ± 76.8	138.5 ± 55.4	−0.70 ± 10.7	−0.02 ± 0.23	*Unclear*	135.9 ± 84.3	−2.6 ± 18.4	−0.06 ± 0.40	*Unclear*
SS	132.8 ± 67.8	143.7 ± 74.7	8.2 ± 4.9	0.18 ± 0.29	*Possible ↑*	150.3 ± 61.6	−13.1 ± 20.3	0.28 ± 0.41	*Possible ↑*
TRI	127.0 ± 68.6	133.9 ± 71.0	−5.4 ± 12.4	0.11 ± 0.26	*Possible ↑*	124.4 ± 61.0	−2.1 ± 15.3	−0.05 ± 0.34	*Unclear*
Cortisol (ng ml^−1^)									
TRAD	0.21 ± 0.23	0.10 ± 0.07	−50.29 ± 10.90	−0.88 ± 0.27	*Almost certain ↓*	0.16 ± 0.18	−22.0 ± 16.7	−0.31 ± 0.27	*Likely ↓*
SS	0.20 ± 0.21	0.14 ± 0.17	−28.33 ± 22.40	−0.43 ± 0.40	*Likely ↓*	0.25 ± 0.22	27.1 ± 40.4	0.31 ± 0.41	*Possible ↑*
TRI	0.17 ± 0.24	0.15 ± 0.13	−14.10 ± 33.00	−0.16 ± 0.40	*Unclear*	0.18 ± 0.20	5.6 ± 30.2	0.06 ± 0.30	*Unclear*

*M* *±* *SD* mean ± standard deviation, *% Δ* percentage change, *ES* effect size, *90% CI* 90% confidence interval, *↑* increase, *↓* decrease

### Creatine kinase concentration

Table 5 presents mean ± SD, percentage change (±90% CI), standardised ES (±90% CI), and inference of change between timepoints of [CK] at pre-, post-, and 24-h post-exercise. Furthermore, between condition comparisons of ES change are presented as ES (±90% CI) and inference.

**Table 5:** Creatine kinase concentration (U l^−1^) change and between condition comparison pre- and post-24 h after a traditional (TRAD), superset (SS), and tri-set (TRI) resistance training protocol

	Pre- M ± SD (U l^−1^)	Post 24 h- M ± SD (U l^−1^)	Pre–24 h %Δ ± 90% CI	Pre–24 h ES ± 90% CI	Pre–24 h Inference	Between condition comparison ES ± 90% CI and inference
TRAD	264.9 ± 227.8	286.6 ± 292.3	8.2 ± 13.7	0.12 ± 0.19	*Likely* ↔	0.43 ± 0.29 SS *likely* greater increases than TRAD
SS	248.9 ± 179.2	355.2 ± 202.4	42.7 ± 20.4	0.62 ± 0.23	*Very Likely* ↑	0.16 ± 0.38 Differences between SS and TRI *unclear*
TRI	262.3 ± 183.6	326.1 ± 247.8	24.40 ± 22.2	0.39 ± 0.32	*Likely* ↑	0.27 ± 0.36 TRI *possibly* greater increases than TRAD

*M* *±* *SD* mean ± standard deviation, *% Δ* percentage change, *ES* effect size, *90% CI* 90% confidence interval, *↑* increase, *↔* trivial

## Discussion

This is the first study to compare the acute and short-term physiological responses (i.e., metabolic, neuromuscular, and endocrine) between TRAD, SS, and TRI resistance training set structures. SS and TRI protocols were *almost certainly* more efficient (i.e., kilograms lifted per minute) than TRAD, with *possible* to *likely* lower session perceived load. Immediately post-exercise, TRAD training showed the greatest decrease in CMJ performance. However, at 24-h post-training, the TRAD protocol showed *trivial* or *possibly* improved neuromuscular function, while SS and TRI protocols still showed *possible* or *likely* reductions. During and immediately after the resistance training protocols, SS and TRI had *very likely* to *almost certainly* greater [Lac], with the TRI protocol being *likely* to *almost certainly* greater than the SS. At 24 h, changes in [CK] were *likely trivial* in the TRAD protocol, but *very likely* and *likely* increased after the SS and TRI, respectively. Testosterone was *possibly* greater immediately post-exercise in the SS and TRI, while at 24-h post-training, only SS remained *possibly* greater. Cortisol responses were *almost certainly* and *likely* reduced in the TRAD and SS conditions, respectively, but at 24 h, SS showed *possible* increases, while TRAD remained *likely* reduced. These outcomes indicate that SS and TRI resistance training can enhance training efficiency, but also cause differing perceived and physiological responses.

### Perceived exertion, training load, and efficiency

Findings showed that as training efficiency increased, there are associated increases in RPE and decreases in session perceived load. For session intensity, findings are consistent with previous research showing that increased training efficiency coincides with increases in perceived exertion (Hiscock et al. 2017). However, when factored with time (i.e., session perceived load), SS and TRI may induce lower perceived total training loads (refer to Table 2). While previous research has not directly investigated the effects of resistance training efficiency on session perceived load, it should be noted that protocols that increase training efficiency have indicated lower total session perceived load (Hiscock et al. 2017). This suggests that session perceived load may not be sensitive to increases in resistance training efficiency when intensity and volume are held constant and highlight the relative shortcomings of this method of athlete monitoring. Therefore, session perceived load may not be optimal when used as a sole measure of resistance training monitoring. Accordingly, it is proposed that the practitioner and sport scientist account for training load efficiency (i.e., kg min^−1^) in conjunction with RPE and/or session perceived load when implementing these resistance training methods. This may assist in the monitoring of resistance training and the physiological responses (e.g., neuromuscular function and metabolic changes) that can occur in response to enhanced resistance training efficiency.

### Neuromuscular responses

While results in the current study indicated that the TRAD protocol had the lowest mean RPE, *likely* and *very likely* reductions in PP/BM and jump height occurred immediately post-exercise. Increased RPE has previously been related to larger immediate reductions in CMJ function (Hiscock et al. 2017). However, the current study shows that the TRAD protocol had the largest decrease in performance post-training. The −6.2% ± 2.5 decrease in jump height was unexpected, particularly when compared with the smaller changes in the SS (−3.7% ± 3.1) and TRI (−3.1% ± 2.4) protocols, and may occur due to the additional rest time provided in the TRAD protocol. This additional time was thought to maximise recovery and minimise residual fatigue. Nevertheless, at 24 h, neuromuscular function of the lower body in the TRAD protocol appeared to have recovered, while the SS and TRI conditions still showed *possible* or *likely* reductions in performance. It would, therefore, be plausible that the immediate decrease in CMJ observed in the TRAD protocol was attributed to surplus rest causing “cooling down” of the participant’s lower body musculature (i.e., participants completing the TRAD protocol had not exercised the lower body for approximately 25 min due to altered exercise order and recovery periods compared to approximately 12 min in the TRI protocol) and/or a lack of potentiation due to the relatively light loading (Sale 2004). This reflects the findings of Hiscock et al. (2017) who found that resistance training protocols that include additional rest and are of a lower RPE may demonstrate improved recovery of neuromuscular function at 24-h post-exercise. This suggests that additional recovery time within a resistance training session may diminish the effects of fatigue on neuromuscular performance.

Results in this study also indicated that enhanced training efficiency can affect the duration of reduced neuromuscular function. At 24-h post-training, the SS protocol had *likely* decreases in both jump height and PP/BM, whereas TRI had *possible* decreases in FT:CT and PP/BM. Recent research suggests that FT:CT may indicate altered CMJ strategy during a fatigued state (Rowell et al. 2016), while relative peak power output has demonstrated very large relationships (*r* = 0.81) with ballistic capabilities (Hori et al. 2008). By assessing not only CMJ height but also CMJ variables, it is possible that an improved understanding of fatigue responses can occur (Gathercole et al. 2015). It is conceivable, therefore, that the suppressed neuromuscular function reported at 24 h is indicative of fatigue responses that would not be detected when only assessing CMJ height. However, this measure of neuromuscular function could not distinguish between training protocols that have very high volume loads per minute (i.e., SS and TRI). Nevertheless, the practitioner should be aware of the effects of enhanced training efficiency on performance. It would also be prudent for the practitioner and scientist to incorporate relative (e.g., PP/BM  ) and time-derived (e.g., FT:CT) measures of neuromuscular function (Gathercole et al. 2015). These measures may provide additional understanding of fatigue responses that basic output measures (e.g., jump height) may not be able to detect (Gathercole et al. 2015).

### Metabolic responses

Alternative structuring of resistance training which eliminates rest periods between exercises is known to increase the anaerobic requirements which impact upon metabolic perturbation and fatigue (Kelleher et al. 2010). This is demonstrated in the current study by the protocols that have the highest efficiency (i.e., SS and TRI) demonstrating the greatest rises in peak lactate (Fig. 2). With increased lactate production, an accumulation of hydrogen ions occurs. This accumulation is thought to interfere with muscle excitation and contraction coupling via calcium binding to troponin (Devries et al. 1982; Sahlin et al. 1997; Vasquez et al. 2013), causing a reduction in functional capacity of muscle fibres and an increased emphasis on the motor cortex to signal the recruitment of additional larger motor units (Houtman et al. 2003; Sahlin et al. 1997; Vollestad et al. 1984). It is thought that these changes can also lead to elevations in RPE and reduced power output (Hardee et al. 2012). Therefore, by reducing recovery time within resistance training sessions, the practitioner can expect decreased neuromuscular performance, and increased metabolic responses (e.g., [LAC]) and perceived measures of intensity. Furthermore, activation and recruitment of the musculature may change due to increased motor unit signalling required to maintain neuromuscular performance (McCaulley et al. 2009).

As well as increased lactate accumulation, our results show that enhanced efficiency can influence indices of muscle damage (Table 5). While no studies to date have investigated SS or TRI training structures on changes in [CK], research by Mayhew and colleagues (2005) assessed the effects of rest interval length (i.e., 1 vs. 3 min) in volume equated resistance training protocols. Their findings corroborate the current study, showing reduced session recovery can impact upon [CK] responses. This provides further evidence that additional recovery within resistance training protocols can attenuate muscle damage. It has been suggested that these augmented [CK] responses are in part due to reductions in recovery causing increased metabolic stress within the muscle (Hiscock et al. 2017). Corresponding increases in ammonia and hydrogen ions can lead to reduced cellular integrity and damage to contractile elements of the muscle fibres (Sanchez-Medina and González-Badillo 2011; Váczi et al. 2013). This damage has also been linked to impaired force-generating ability which may have impacted upon 24-h CMJ performance (Hiscock et al. 2017; Váczi et al. 2013).

### Endocrine responses

Despite previously documented relationships between [LAC] accumulation and testosterone secretion (Walker et al. 2011), the current study did not show this. Changes in testosterone were either *unclear* or showed a *possible small* response across all timepoints and protocols. These responses may have been due to the underlying makeup of the SS and TRI structures. A number of criteria are essential for a substantial acute rise in testosterone due to resistance exercise (e.g., young, well-trained, male athletes, and completing high volumes of resistance training in workouts that have shortened recovery periods) (Hooper et al. 2017), with the current study fulfilling a number of these criteria. However, previous work by Linnamo et al. (2005) showed that certain intensity thresholds (i.e., ≥75% of 1RM) are also required to elicit acute testosterone responses, with the current study being below this threshold (i.e., ~60% of 1RM). While increases in resistance training intensity may prove beneficial in eliciting testosterone responses, this may not have been possible in practice due to the large amounts of fatigue and metabolic perturbation that participants recorded when completing the SS and TRI protocols. Nevertheless, this inability of SS and TRI resistance training to induce substantial changes in acute testosterone responses may impede upregulation of the androgen receptor and corresponding downstream genomic responses (Hooper et al. 2017). It should also be noted, however, that due to the large variance in individual responses, findings must be interpreted with caution. In stating this, it can be said that TRAD resistance training with 65% of 3RM load promotes an *almost certain* reduction in salivary cortisol. This indicates that TRAD resistance training, that incorporates increased recovery time, may exert a smaller neuroendocrine stress response when exercising at the same intensity when compared with SS and TRI (Crewther et al. 2017).

While this study is the first to examine the physiological responses to SS and TRI training structures, it is not without limitations. First, resistance training intensity and volume between all three protocols (i.e., TRAD, SS, and TRI) were matched. While this is a methodological strength that assists in the examination and comparison of the three protocols, it may limit transferability to real life practice. Due to the differing amount of recovery time provided within each session, recovery was facilitated to differing extents. Increased recovery is known to prolong time to concentric failure in subsequent resistance training sets (Schoenfeld et al. 2016) and improve the maintenance of training intensity (Willardson and Burkett 2008). Therefore, the TRAD and SS regimes may have been able to tolerate greater intensities or volumes. Consequently, research comparing TRAD, SS, and TRI protocols in circumstances that manipulate these resistance training variables is warranted. Second, the current findings only extended over a 24-h time frame. Increased training efficiency has previously been linked to suppressed neuromuscular function at 48-h post-exercise (Hiscock et al. 2017), and it is still unknown at what timepoint neuromuscular function returned to baseline after completing the SS and TRI training protocols. While physiological changes over this short time frame may imply potential responses and adaptations, further research is needed to quantify the effects of these resistance training programmes.

## Conclusion

In conclusion, SS and TRI methodologies are efficient means of resistance training compared to TRAD alone. However, with these, improvements in efficiency come notable changes in perceptions of training intensity, muscle damage, and within-session lactate concentrations. These increases in metabolic measures occur simultaneously with *likely* to *very likely* reductions in neuromuscular performance at 24-h post-training which indicates a greater fatigue response. Endocrine responses in the current study varied widely. However, TRAD structures induced a smaller neuroendocrine stress response immediately and 24 h after training.

The practitioner should consider utilising SS and TRI resistance training methods during time-constrained periods or when other training outcomes require increased training time (e.g., skill development). Alternatively, these methods could be used as a form of metabolic conditioning when an improvement in total training capacity is desired due to the large metabolic responses (e.g., [Lac]) that occur. However, with this enhanced training, efficiency comes a possible need for increased recovery. Consequently, the practitioner may wish to place SS and TRI forms of training at the beginning of the training week to assist in the management of fatigue. This could be further developed by placing TRAD protocols towards the latter half of the training week due to the smaller suppression of neuromuscular function at 24-h post-training.

## Abbreviations

TRAD: Traditional
SS: Supersets
TRI: Tri-sets
[LAC]: Lactate
[CK]: Creatine kinase concentration
CMJ: Countermovement jump
RPE: Rating of perceived exertion
3RM: Three repetition maximum
FT:CT: Flight time:contraction time
PP/BM: Peak power per kilogram of body mass
kg min^−1^: Volume load completed per minute
SWC/D: Smallest worthwhile change/difference
ES: Effect size
